# Polymorphisms in *COL4A3* and *COL4A4* genes associated with keratoconus

**Published:** 2009-12-20

**Authors:** Mirna Štabuc-Šilih, Metka Ravnik-Glavač, Damjan Glavač, Marko Hawlina, Mojca Stražišar

**Affiliations:** 1 Eye Hospital, University Medical Centre, Ljubljana, Slovenia; 2 Department of Molecular Genetics, Faculty of Medicine, University of Ljubljana, Slovenia

## Abstract

**Purpose:**

Alterations in collagen type IV, alpha-3 (*COL4A3*) and collagen type IV, alpha-4 (*COL4A4*) genes may be responsible for a decrease in collagen types I and III, a feature often detected in keratoconus (KC). To evaluate the significance of alterations in *COL4A3* and *COL4A4* genes in KC patients, we screened both genes and estimated the significance of polymorphisms in Slovenian patients with KC.

**Methods:**

The study included 104 unrelated patients with KC and 157 healthy blood donors. Diagnosis was established by clinical examination, electronic refractometry, and keratometry. DNA was extracted from blood, and gene exons were amplified by PCR. Non-isotopic high-resolution single-stranded conformation analysis (SSCA) was used to screen *COL4A3* and *COL4A4* genes, and migration shifts detected by SSCA were subsequently sequenced. For statistical evaluation, control blood donors were chosen according to age, sex, and not having blood relationship. Neither patients nor control blood donors chosen for statistical analysis were in blood relationship. We used Fisher’s exact test for statistical evaluation, with p<0.05 considered significant.

**Results:**

We detected eight polymorphisms in the *COL4A3* gene and six in the *COL4A4* gene. Allele differences in D326Y in *COL4A3* and M1237V and F1644F in *COL4A4* are significantly distinctive of KC patients (Fisher’s exact test, p<0.05). When analyzing different genotypes under three models (dominant, recessive, and additive), we established that P141L, D326Y, and G895G in *COL4A3* and P482S, M1327V, V1516V, and F1644F in *COL4A4* have significant differences in genotype distribution between KC patients and the control group.

**Conclusions:**

This is the first mutational screening of *COL4A3* and *COL4A4* genes in KC patients to establish the status of these genes and compare them to a control population. Analysis of *COL4A3* and *COL4A4* revealed no mutations related to KC patients, but specific genotypes of seven previously described polymorphisms are significantly associated with KC under dominant, recessive, or additive models. Differences in the expression of type IV collagen in previously published data about chromosomal instabilities in the regions in which the analyzed genes were mapped and our data indicate a probability that some of the polymorphisms we detected could be related to KC.

## Introduction

Keratoconus (KC) is a noninflammatory progressive thinning disorder of the cornea that leads to progressive mixed myopic and irregular astigmatism [1]. The estimated incidence of KC is between 1 in 500 and 1 in 2,000 in the general population [1]. KC occurs in all ethnic groups, with no significant gender difference. The age of onset is puberty, and KC is progressive until the third to fourth decade of life when it usually arrests. It is the major cause of cornea transplantation in developed countries. Although the cause of KC is unknown, there are several lines of evidence suggesting a genetic component. These include a positive family history in 6–10% of KC cases [1,2] and its higher concordance rate in monozygotic twins [1,3]. Although the disease has been reported to exhibit familiar patterns and an autosomal recessive mode of inheritance has been postulated, most cases appear to be sporadic [4,5] Hereditary KC is inherited dominantly or recessively, but families are frequently diagnosed with autosomal dominant, which presents incomplete penetrance of the disease and variable expressivity [5]. The underlying biochemical processes and their cause remain poorly understood. By far the most common presentation of KC is as an isolated sporadic disorder, but a positive association between KC and many conditions has been suggested, including atopy, eye rubbing, wearing hard contact lens, and cardiovascular disease (especially mitral valve prolapse) as well as some rare genetic disorders, connective tissue disorders, pigmentary retinopathy, Marfan’s syndrome, Noonan’s syndrome, Apert’s syndrome, Ehlers-Danlos syndrome, and Down syndrome [1,6].

The major protein in the cornea is collagen, and several types of collagen have been identified by biochemical and immunochemical methods [7]. Corneas from patients with KC contain reduced amounts of total collagen proteins, [8] and alterations of the extra cellular matrix and basement membrane are characterized mostly by a decrease in types I and III [9]. The changes in the orientation of collagen molecules, which are followed by rearrangement of collagen fibrils, also alter the shape and transparency of the cornea [10,11]. A knockout mouse model has shown that disruption of the genes encoding α1 (*COL8A1*) and α2 chains (*COL8A2*) of type VIII collagen leads to structural changes similar to the clinical presentation of keratoglobus [12]. KC has not been associated with mutations in type VIII collagen genes [13], although a relation between *COL8A2* mutations and dystrophic corneal disorders has previously been reported [14,15]. Results from imunohistochemistry, in situ hybridization, and expression arrays show that several other types of collagen are differentially expressed and have an active role in wound healing processes. Collagen molecules that are differentially expressed in keratoconus corneas are types XII, XIII, XVIII, and XV, but there are no known relations between mutations and expression levels for those genes [16,17]. Upregulation of collagen type XV and downregulation of collagen type IV in KC corneas, observed by Bochert et al. [18] and Stachs et al. [19], showed the putative role of those types of collagen in KC. Types XIII, XV, and XVIII collagen were found to be expressed in basal corneal cells and may have a role in the adhesion of the corneal epithelial cells to each other and to the underlying basement membrane [16,19].

Type IV collagen is found only in basement membranes where it is the major structural component. Mariyama et al. [20] mapped the collagen type IV, alpha-3 (*COL4A3*) and collagen type IV, alpha-4 (*COL4A4*) genes to the same region, 2q35-q37, but on opposite strands and transcribed in opposite directions [21]. The *COL4A3* gene spans 250 kb and consists of 51 exons; the *COL4A4* gene is shorter, spanning 113 kb and consisting of 48 exons [20,22]. *COL4A3* and *COL4A4* are two of six α chains that form heterotrimeric type IV collagen molecules [20,23,24]. Type IV collagen is expressed in corneas and implicated in Goodpasture and Alport syndromes, which are often accompanied by eye abnormalities, but their involvement in eye  disorders is still unknown [22,24-26]. *COL4A3* has already been implicated in the pathogenesis of polymorphous corneal dystrophy-3 [27,28], and both genes are reported to be differentially expressed in keratoconus corneas [18,19]. Results from the study published by Stachs et al., favored collagen type IV as a candidate gene in keratoconus pathogenesis [19]. Because a change in the expression levels of collagen type IV α-3 and α-4 chains were observed in corneas affected by KC, we investigated whether there are alterations in *COL4A3* and *COL4A4* related to KC patients.

## Methods

### Patients

The genetic study included 104 unrelated patients with KC and 157 healthy blood donors as a control. After examination of the patients (clinical examination, electronic refractometry, and keratometry) and precise personal anamnesis, an unrelated cohort of patients diagnosed with KC was selected for this study. We excluded patients with other ocular diseases that could influence the interpretation of the results: blepharoconjunctivitis, keratitis, opacifications of the lens, changes of the macula, and cup/disc ratio  (C/D) of the optic nerve of 0.3 or more. One hundred and four patients, 65 males and 39 females, were included in this study after informed consent had been obtained and after determination of the diagnostic and other criteria. All the patients included in the study had no other diagnosed disease. The patients’ ages were from 20 to 67 years (mean±standard deviation [SD] 39.1±8.2 years). For the control population we used peripheral blood taken from 157 blood donors collected at the Blood Transfusion Centre of Slovenia (57 women, 100 men; mean age ± SD 37.2±10.2 years). Blood samples from patients with KC and from healthy Slovenian blood donors were in the form of anticoagulated blood. Blood from KC patients and controls was obtained from the median cubital vein, on the anterior forearm in 3 ml vacuum blood collection tubes with EDTA K3 (Laboratorijska tehnika). Blood was stored in collection tubes at -20 °C until the DNA was isolated. The control group was selected on the basis of age, nationality, and gender comparable with the KC patients. There were no blood relations among individuals in the control group or between individuals in the control group and  individuals in the KC group, and control individuals had not been diagnosed with KC. The National Medical Ethics Committee of the Republic of Slovenia approved the study.

### DNA extraction and mutational screening

Genomic DNA was isolated from peripheral blood lymphocytes by salt precipitation. After the blood samples were thawed, saline-sodium citrate buffer (Merck) was added, mixed on Vibromix (Tehtnica), and the samples centrifuged (12,000 rpm for one minute, centrifuge 5415R; Eppendorf). The top portion of the supernatant was discarded and saline-sodium citrate buffer (Merck) was added, mixed, and again mixture centrifuged under same conditions. Then was supernatant discarded and pellet re-suspended in a solution of sodium dodecyl sulfate detergent (10 % SDS; Sigma-Aldrich) and 5 µl of proteinase K (20 mg/ml H_2_O; Sigma-Aldrich). The mixture was incubated at 55 °C for 1 h (Thermomixer comfort; Eppendorf). After incubation was DNA treated with a phenol/chloroform/isoamyl alcohol solution in ratio 25:24:1 (Sigma-Aldrich).  After centrifugation (12,000 rpm for 1 min, centrifuge 5415R; Eppendorf) was the aqueous layer removed to a new micro centrifuge tube (Costar) and two consecutive DNA ethanol precipitations followed; first one with 100 % and second one with 80 % ethanol (Merck). DNA was re-suspended in 10:1 Tris-EDTA buffer (Sigma-Aldrich) between both precipitations. After the second precipitation the pellets were dried at room temperature followed by addition of 10:1 Tris-EDTA buffer (Sigma-Aldrich). The mixture of DNA and Tris-EDTA buffer (Sigma-Aldrich) was re-suspended with mixing and incubation at 55 °C overnight (Thermomixer comfort; Eppendorf). Amplifications of *COL4A3* and *COL4A4* were performed by PCR. For the PCR reaction we used the primers (Operon) previously described by Heidet et al. [29] (*COL4A3*; Table 1) and Boye et al. [22] (*COL4A4*; Table 2).

**Table 1:** Oligonucleotide primers and PCR temperatures used for single-stranded conformational analysis and sequencing of the *COL4A3* gene [29].

***COL4A3***	**Sense primer (5'→3')**	**Antisense primer (5'→3')**	**Length (bp)**	**Annealing temperature (°C)**
EX 1	CGGACTCGCCCAGGCTCTGA	GACGCGTGGAGGAGGGATG	176	62
EX 2	AACAAAACCCTTTCTCTT	AAGCAGTATTAGGGTTTGTT	113	49
EX 3	TGTGTGTTTCTCACCTCGT	GATTTTCCAAGCTTGCAG	151	54
EX 4	TTTCTTTTTTCACTTGAATCT	ACGATCAGGGTGGACTG	99	50
EX 5	CCCCCTCCTTTTTCCTATGT	TTTCTAGCTACGGATTTTTC	102	45
EX 6	CCTCATTGAGACTTGTTCT	TCATCTTCTGTGTGAAAAGT	116	42
EX 7	AATAATAAGAAACTTTGTATGT	GGGAATTAGGCATGCAAA	106	49
EX 8	GTTGTTCATAGGTTGCTTTT	TCAGTGACAGCATTCCAC	83	46
EX 9	GATGTTTGATGAACTTCTTC	ATAGGGACCTTCTCTGAA	134	52
EX 10	TACTCTTATTCTTCTCTCAA	CTGTAGCAAGGATGACT	117	49
EX 11	GTGATTTTCATTTGTGGATT	AGCTGTTACATCATATGAACT	93	48
EX 12	AATAATTTGGTTTTGTGTT	CCTGCTAATAAAACATAGTA	100	44
EX 13	ACTCCTGAGTGTTTTTGT	TAATCATAAAATCGCAGA	126	49
EX 14	TTGTAACAATGTTGAACTGT	ATGGGGACAATATAACTTTA	124	50
EX 15	ATAAAATTTGACATGGCTCT	GACTAATCAAAACTGCACAT	133	49
EX 16	TTTCATGTTTTTGATTTGTT	TGACATTTTTACTACCTCCA	116	46
EX 17	GACCCATTTCTTTTTGTTCT	AAAATAGGCTATTAGGGAGA	110	48
EX 18	CACAATTTGTAAATGTCTT	GATATTGTCTTTAATCACAC	94	46
EX 19	TCTGTATTTGTTTCTTTCTC	AAATGCTTTAGGAAGAAAT	141	52
EX 20	TTATATCTTTCTAAGCCATT	CCTTTGTAATAGCATTTCTA	125	47
EX 21	TCTCCATTGTGCAATTTTTA	CTAAGCTGTGAGGAGGGTTT	367	53
EX 22	ATTGTCTTTGGTGCTGTAT	GGCTTATCCTAATACAACAT	156	49
EX 23	AAGTAATGCTAGTATGCTCTC	TGTGCTTGCAAAAACACT	162	49
EX 24	TAGTTAATAATTCGTTGA	AAGATTTAAAAACATGAA	121	44
EX 25	ACAGATTCATTTGTGTACTA	GAGGGTAAAGTTGCTAAATA	234	54
EX 26	ATTCAAACACATTCCTGT	GGACTGGAAAGAAAACTAA	219	51
EX 27	ATCTTATGACCACAAATTTC	CAGATTTGGCAGAGGATA	142	54
EX 28	AGATGCATATGTGTATTTGT	CTTCTAAATATCCACAACAA	182	44
EX 29	CTAATCCTACAACAATGTTT	TTCTGTGATAGCTTGAATTT	164	47
EX 30	ATAGTAATAACACAATTTCT	GAGAAAAGTAATGACACT	209	45
EX 31	CCTGGGTATATACTTGTGCT	ATGTCTCCTGCCCTTCTGG	191	52
EX 32	GGAAAGCATTTGTGGGTTA	ACAGAGCCACCTTAAGAAGA	276	52
EX 33	TGCTTTGTGTTAATTTGTTT	TCCTGCTATTTAGAAAGACA	149	52
EX 34	AAGGACCTGATGTTGTTACT	TCTGATGTCCTGATTCCA	202	52
EX 35	TTCTTGTTAATACCTGGTTT	TGATATTTTTCTATTTGAGA	160	49
EX 36	CAGGGCAATAACTACTTA	GCTCATAACAGGACCTTA	146	50
EX 37	TACTCTATGTTTTCCCCCTA	TCCACCACTAAAATGTAAAT	206	52
EX 38	TATGAGAATTTTAAAGGTAT	TCCAGCTTTTAGAATTGTAA	195	48
EX 39	GGTGATCTTTTTTCTTCCTT	CCCACATGAAAAGGAAAAAG	160	50
EX 40	GGGGTTTTGGGTTTTTTT	ACGGATCAAAGATAATGAGCA	156	52
EX 41	CAATTATTAACATGCCAAGA	TACATTAGGACAGGGAAGAA	112	50
EX 42	AAAGAAACTTATTAAGCCTT	TTGTTATTTTATGCTGTTTA	253	52
EX 43	ATACTGACAGACTTTTCAT	TAATAATGAGTCAAAATAAT	191	50
EX 44	GTTTTGCTCCCTTTATTTGA	ATATAAAGAGCAATGCACAA	129	51
EX 45	GGAAACCCATTGATCTAAGT	ACCTTTCTTCATTGACAGCA	164	51
EX 46	TGAGGCCATCATCTTCTTCT	TCCTAGTGATCCAAGTCAAT	199	50
EX 47	CCACCTTACTTTTCATCCTAT	ACTTCTTCGGTGAGGAAAC	190	59
EX 48	CTTTGAAAAAACGAGTTTAAG	TTACAATCTGCATGTGGAA	324	61
EX 49	CTAGTAACGATGCTGAAAATAAC	TCACTTGGTCCCATTGTAA	284	54
EX 50	TTCCCTTGTAATGGAATGAAA	CACATTTTACCCAGCACAAT	271	52
EX 51	AACCCCAATGGACAGAGTGTT	TGAATAGTTCTGCAATTGAGT	272	63
EX 52	CAGCAAAAATTCCCTTTTATG	TGTTCTTTAGGATGAAAAAT	190	47

In the table, Length represents length of the PCR product in base pairs (bp) and Annealing temp represents the annealing temperature of the primers used for PCR reactions.

**Table 2:** Oligonucleotide primers and PCR temperatures used for single-stranded conformational analysis and sequencing of the *COL4A4* gene [22].

***COL4A4***	**Sense primer (5'→3')**	**Antisense primer (5'→3')**	**Length (bp)**	**Annealing temperature (°C)**
EX 2	TCTGGAAGAGAAGACTGGCA	AAGCAGGCAATCACACTGA	153	54
EX 3	TGTTTAAATTAATCTGCGTT	GCAACCAGAGCTAGTG	105	48
EX 4	CGATGAGTACTGGTATACTA	ATGCTGCCCATGTTGGTCTT	152	50
EX 5	ACCCCCATTTCTTTTTAATC	GGTGAGTCTTTCATGTGAAT	208	54
EX 6	TCTCTTTGTTTTATTTTCTG	GATGAGTACTTCTGCCTTTT	127	47
EX 7	TTTCGCAAAAATGCTTCACT	CCACAGGGCCTGTTCACTTA	211	60
EX 8	TACTGAAATGGTAATACGCT	CATGGGCTTACCTATTTGGA	184	48
EX 9	TGTGTGGACTTAAAGCGATG	TAGAGCCTGCTCAGGAGACT	96	53
EX 10	TTGGGTAACAGATGCACTGA	AAGGGATCACATCAGCAGTG	129	55
EX 11	TTGTGTTTTTTTCTCCCTTG	TTTCATTGTTCAGGGCTCTA	109	50
EX 12	AGCCAGAAGTCTTAATTGCT	TCACCATTTGCTCCTCAGAG	156	54
EX 13	GGGTGGAAACCTTCAAAACA	TACTTTCCAAGGTGACATAT	179	50
EX 14	GGAGATGGAATTCAGTATGT	AAAGACCATGAGAAATAACA	197	53
EX 15	CCCCTCTAAATGTTGTCATC	TTTGAGCTTGTGGGACTACT	180	54
EX 16	AATGATGCACTGAGCTGGTT	GCACGCAACAGTACAACTTC	200	53
EX 17	ATTTGTCACCCCGTCACTTT	GAATGATTCCTGGCAATACT	201	50
EX 18	CCAGGCAACATGAGTAAAAT	TGGAGGAACTGAATAGGAAC	155	50
EX 19	TGCACATACCATTTGTTTAT	CCAGGGCACATCAGGGCATC	175	50
EX 20	TTCTTCTACAGAGACGTTT	TGCTAATGGATATGAATAAG	259	52
EX 21	TATAGAAGACAGTCAGAAAA	TAGAAATTCTACCTTTGGTG	181	50
EX 22	AAATATGACAAATCTGCCAT	GGAAAGATGACTGGTAAGAG	227	50
EX 23	TGATCCATCACAATTAACCT	CAGGGAGTTAAGTGATTGAT	149	53
EX 24	ACTTTACCCTCTGCTGATAA	GGGAAATAGTTGTTTGTATG	223	50
EX 25	GACATTCAGTGGTTGGTAAT	TAAACACTTGTACCCCAAAG	280	60
EX 26	TCAGTTATGTGAATGCCGCT	TGGGAAGTATATAAGACAGT	147	50
EX 27	TGAGTCTGTGTTTTGTTTTT	AAAAAAAAAAACCTCAC	210	52
EX 28	ATTGGTTCTATACTTGCACA	TCTATGCACCAAAAGGACAG	309	54
EX 29	TGGGCCATCTGTATAGTTTT	TAATAGTAAGTAGGGTAAGC	269	57
EX 30	GCCTTCACACACTGTGGTCA	ATGGGAGGACATCATGGAAA	240	55
EX 31	TCCTAAAACTTTATGCTCTC	TCAAATACCAGAAACAAATG	221	53
EX 32	CCTGTTCATTTTGTTCTTGC	TGTCAACTTATTTGATATGG	187	57
EX 33	TTTCAGCAGAGACCTGTAAC	AAGAACAGAAAGGTTTTATT	271	52
EX 34	GTTGTGCATGTGCCATTTGT	GATGGCTTCTGTATCTCC	154	50
EX 35	TGAGACCAAATTAAATTGTC	TCATTGCCAGCTAGAAGTAA	210	52
EX 36	CAAACGGCAACTCTGATGTT	AGTGCTCAGGAAGTCTCCAG	183	55
EX 37	TATCTGGCCATCTGCAAAAC	TTGTGGGATGGGCTTCATTT	173	55
EX 38	GCGTTTGTGGCTAGAGTGAG	GAACCATGGACTGAAGCTCAG	190	57
EX 39	AGGCACTATAACAGGGACAAGA	GGAGTAACGTAAACCTTCCA	256	60
EX 40	ACCTTCCAAATGCAATGAGG	CATCCTTTGTCATGATTCTCTC	184	53
EX 41	TTTTTGTCTCTTCTCTGTGG	AGTTATTCACATATTACTTA	218	48
EX 42	GCCCTCATTTTTATGTTTTG	GTTGGAAGCTCACCTGGAAG	153	54
EX 43	GACTGGCCTCGTTTG	TTAATATCCTTACAGCACCC	180	50
EX 44	ATTACACAAGCGGTGATTCC	TGGCTCCTTCTGGTCCTCTC	118	56
EX 45	CACCAGCATCATAAACTT	AGGTTTACAGTGTCAGAGAA	186	53
EX 46	AGTGCCAGAACAGAGGTGCT	GGAGATGGGCGATCCTGTA	297	57
EX 47	ACACCAGCTGTCTCTTCTTC	TGAATGAGCCAGGGTTT	353	57
EX 48	GTGTGTGTCTGAGCCCTAAT	TGGTGAATTTCGCATTCT	322	50

In the table, Length represents length of the PCR product in base pairs (bp) and Annealing temp represents the annealing temperature of the primers used for PCR reactions.

**Figure 1:**
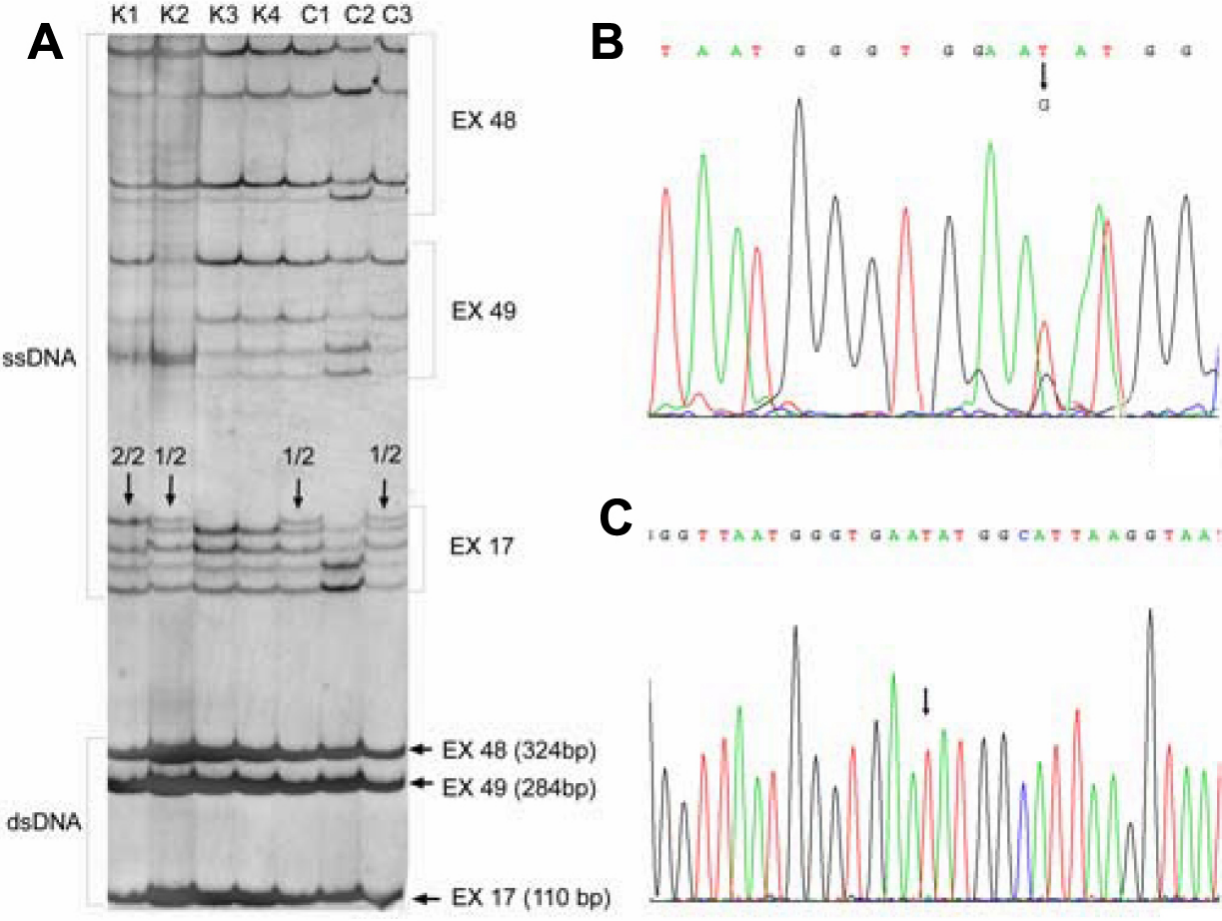
**A**: Three different PCR–single stranded conformational analysis patterns on one gel, representing *COL4A3* exons 17, 48, and 49. PCR fragments were loaded in succession from shortest to longest PCR fragment at 30-min intervals. Exons 48 and 49 did not show any differences in elution shifts; in exon 17, the different patterns were subsequently sequenced. **B**: Partial sequence of exon 17 with heterozygous substitution D326Y (976GT). **C**: Partial sequence of exon 17 with homozygous substitution 326Y (976TT). K1 to K4 marked patterns are patterns of three exons (17, 48, and 49) of *COL4A3* from keratoconus patients, multiplied by PCR and analysed with SSCA. C1 to C3 marked patterns are patterns of three exons (17, 48, and 49) of *COL4A3* from controls multiplied by PCR and analysed with SSCA. '1/1', '2/2' denote different genotypes at position 976 (1/2 being GT and 2/2 being TT) in *COL4A3*. GG genotypes, being  '1/1’ genotypes, are unmarked. Single stranded DNA (ssDNA) and double stranded DNA (dsDNA) patterns are marked on the side of the SSCA gel.

**Figure 2:**
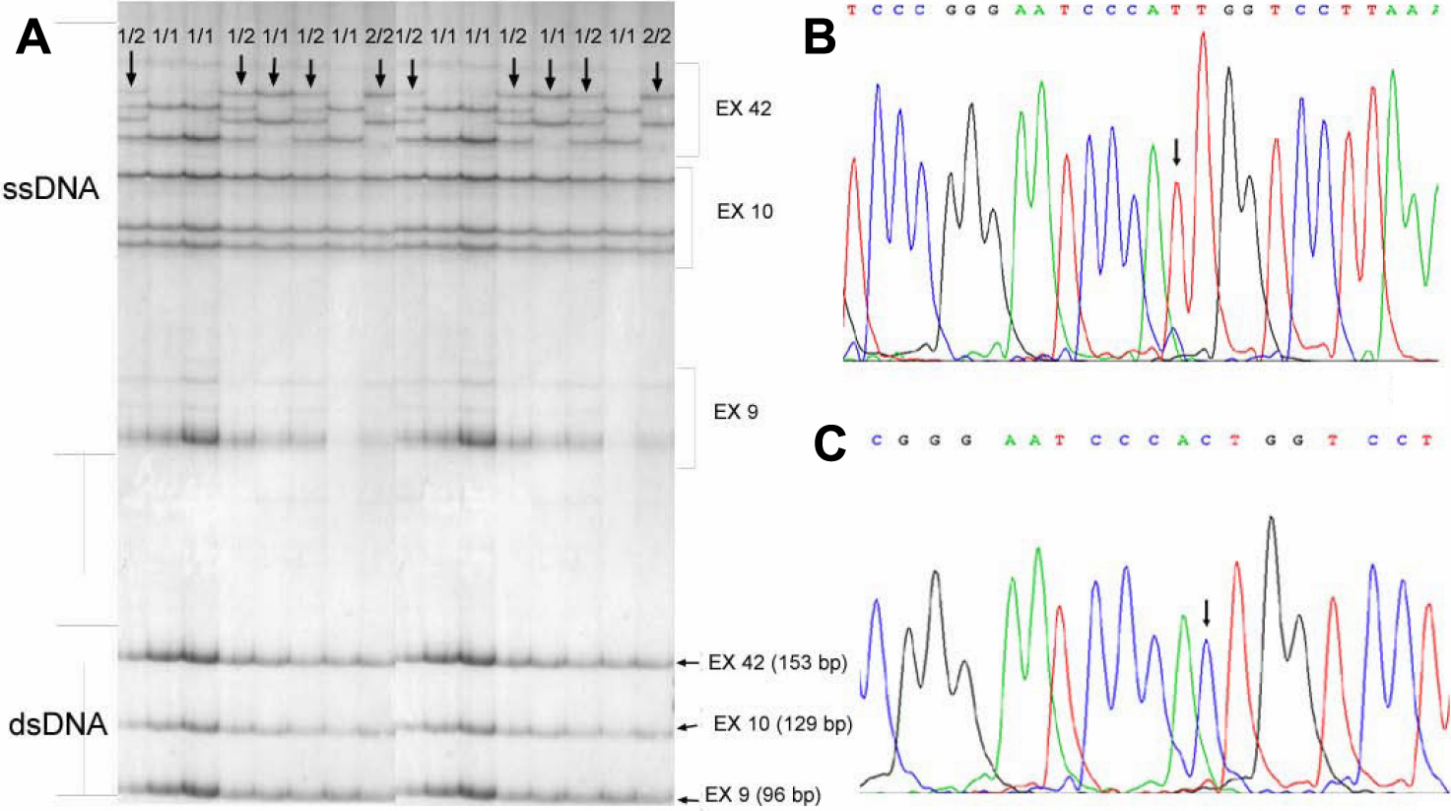
**A**: Three different PCR–single stranded conformational analysis patterns on one gel, representing *COL4A4* exons 9, 10, and 42. PCR fragments were loaded in succession from shortest to longest PCR fragment at 30-min intervals. Exons 9 and 10 did not show any differences in elution shifts; in exon 42, different patterns were subsequently sequenced. **B**: Partial sequence of exon 42, made with reverse primer, showing homozygous substitution 1327M (3797AA). The position marked on reverse sequence is 3797TT. **C**: Partial sequence of exon 42, made with reverse primer, showing homozygous substitution 1327V (3797GG). The position marked on reverse sequence is 3797CC. '1/1',’1/2’ and '2/2' denote different genotypes at position 1327 in *COL4A4*. Single stranded DNA (ssDNA) and double stranded DNA (dsDNA) patterns are marked on the side of the SSCA gel.

Screening for changes in PCR products was performed with single-stranded conformation analysis (SSCA) for each PCR fragment of a given set of samples from patients and healthy blood donors. Large glass plates (35×40 cm) were used to obtain maximum sensitivity. The shorter plate was coated with Repel-Silane (Merck). The longer plate was coated with Bind-Silane (20 ml of g-methacryloxypropyltrimethoxysilane, 5 ml of bi-distilled H_2_O, 5 ml of 100 % ethanol; Merck), then warmed to 50 °C for about 30 min and cooled to room temperature. A 3 ml portion of PCR product was mixed with 10 ml of loading buffer (95% formamide, 5 mM NaOH, 0.1% bromophenol blue, and 0.1% xylene cyanol, Sigma-Aldrich). Samples were heated to 95 °C for 2 min and then cooled in ice water. Each PCR product mixed with loading buffer was loaded onto polyacrylamide gels (35×40×0.04 cm). Combs with 96 teeth were used for loading samples and 10% acrylamide gels (Merck) with 2.6% bis-acrylamide (Merck) to analyze multiple exons at once. Gels were run at 4 °C in 1× TBE (50mM Tris-borate, pH 8.3, 4 mM ethylenediaminetetraacetic acid; Merck). For DNA visualization, we used the optimized method of Heukeshoven and Dernick [30] with most phases at 55°C. Silver staining (Merck) was performed on thin gels (0.4 mm) fixed on the larger glass plate. Samples with different migration shifts were chosen for sequencing, which was done with a BigDye Terminator Ready Reaction Mix (Applied Biosystems). Sequences were purified, dissolved, and analyzed on an ABI PRISM 310 Genetic Analyzer (Applied Biosystems; Figure 1 and Figure 2).

### Deviations of the Hardy-Weinberg equilibrium

Deviations of the Hardy-Weinberg (H-W) equilibrium were calculated with the χ^2^ online test with 1 degree of freedom (DF=1) for each polymorphism found in KC patients and control groups. By the use of a χ^2^ table, with DF=1, the limits for maintaining a null hypothesis (that the observed data has Hardy-Weinberg proportions) were obtained. If the result equaled or was less than 0.05 (5% limits), we concluded that there was no statistical deviation from the Hardy-Weinberg equilibrium in our data (Table 3).

**Table 3:** Data about observed polymorphisms in keratoconus patients and controls with calculated deviation from Hardy-Weinberg equilibrium.

**Polymorphism**	**Exon**	**dbSNP ref ID**	**Hardy-Weinberg CHI (p-value)**	
			**Cases**	**Controls**
***COL4A3***				
G43R	2	rs13424243	0.2127 (p>0.2)	0.2928 (p>0.2)
P141L	7	rs10178458	2.1005 (p<0.2)	18.333 (p<0.0001)
E162G	9	rs6436669	1.6271 (p>0.2)	0.9575 (p>0.2)
D326Y	17	rs55703767	3.6385 (p<0.1)	11.6848 (p<0.001)
H451R	22	rs11677877	0.6282 (p>0.2)	0.8912 (p>0.2)
G484G	23	rs34019152	0.2127 (p>0.2)	0.4525 (p>0.2)
P574L	25	rs28381984	4.7767 (p<0.05)	0.8502 (p>0.2)
G895G	33	ref*	0.2376 (p>0.2)	5.8157 (p<0.02)
***COL4A4***				
P482S	21	rs2229814	0.5769 (p>0.2)	30.0822 (p<0.0001)
G545A	23	rs1800516	0.1261 (p>0.2)	0.2928 (p>0.2)
G789G	28	rs56247709	5.0335 (p<0.025)	0.2478 (p>0.2)
M1327V	42	rs2229813	12.1981 (p<0.001)	9.1888 (p<0.01)
V1516V	47	rs2228555	0.6683 (p>0.2)	8.5342 (p<0.01)
F1644F	48	rs2228557	0.4009 (p>0.2)	1.3937 (p>0.2)

dbSNP ref ID: identity numbers for observed variants; ref*: polymorphism is not listed in dbSNP, but was reported by Wang et al. [34]. Hardy-Weinberg CHI (p-value): calculated chi values according to our data for cases and controls separately, and deviation between observed and expected numbers. When p-value equals or is less than 0.05 (5%) limit, then there is no statistical deviation from the Hardy-Weinberg equilibrium.

### Associations between allele and genotype frequencies

The magnitudes and directions of associations between the polymorphisms found and KC patients were determined using Fisher’s exact test with a two-sided p value. Fisher’s exact test was chosen because it is based on exact probabilities from a specific distribution and is the preferred tool over the χ^2^ when comparing small data samples and a large sample approximation would be inappropriate. A two-sided p value was calculated to determine the significance of the relationship, and a value of p<0.05 was considered statistically significant. Significant relationships for each allele or genotype group between KC patients and the control group are summarized as odds ratio (OR) and relative risk (RR; Table 4).

**Table 4:** Allele frequencies and their significances in *COL4A3* and *COL4A4* polymorphisms between keratoconus patients and control population.

***COL4A3* polymorphism**	**Allele**	**Cases (n=208)**	**Controls (n=314)**	**p-value**	**OR**	**RR**
G43R	127G	199	301			
	127C	9	13	1.0000		
P141L	422C	172	261			
	422T	36	53	0.9059		
E162G	485A	174	262			
	485G	34	52	1.0000		
D326Y	976G	199	187			
	976T	9	127	<0.0001	15.017	7.790
H451R	1352A	193	292			
	1352G	15	22	1.0000		
G484G	1452G	199	298			
	1452A	9	16	0.8349		
P574L	1721C	118	166			
	1721T	90	148	0.4195		
G895G	2685A	137	227			
	2685C	71	87	0.1208		
***COL4A4* polymorphism**	**Allele**	**Cases (n=208)**	**Controls (n=314)**	**p-value**	**OR**	**RR**
P482S	1444C	117	182			
	1444T	91	132	0.7183		
G545A	1634G	201	301			
	1634C	7	13	0.8168		
G789G	2367G	200	302			
	2367A	8	12	1.0000		
M1327V	3979A	74	182			
	3979G	134	132	<0.0001	0.4005	0.5738
V1516V	4548A	119	184			
	4548G	89	130	0.7861		
F1644F	4932C	136	163			
	4932T	72	151	0.0028	1.750	1.409

Cases: number of alleles found in keratoconus patients, Controls: number of alleles found in healthy blood donor population, n: number of all alleles, p-value: two sided p-value calculated with Fisher’s exact test for determining the significance between differences in alleles found in keratoconus patients and controls for each polymorphism, OR: odds ratio, RR: relative risk. OR and RR are shown only for polymorphisms for which allele differences are significant (p-value less than 0.05).

The significance of genotype frequencies for each polymorphism found in the two-tested groups (patients and controls) was tested in two models, dominant and recessive. A dominant model was constructed on the basis of a presumption that at least one allele would be changed. We therefore combined the number of heterozygous genotypes with the number of homozygous genotypes for each polymorphism genotype and analyzed whether the representation of genotypes was significantly different between cases and controls for each polymorphism (Table 5). A recessive model was constructed on the basis of a presumption that both alleles would be changed. Therefore the number of homozygous genotypes against combined heterozygous and homozygous genotypes for another allele were compared for each polymorphism and whether the representation of genotypes was significantly different between cases and controls for each polymorphism was analyzed (Table 5). An additive model was constructed to test the significances between KC patients and the control group for all genotypes in detected polymorphisms (Table 6). For statistics we used the Fisher’s exact test, and when the two-sided value was less than 0.05, the results were summarized as an OR and RR. All statistical analyses were performed using SPSS ver.14 (SPSS Inc.).

**Table 5:** Genotype representation and associations under dominant and recessive model between keratoconus patients and controls.

***COL4A3* polymorphism**	**Genotype**	**Cases (n=104)**	**Controls (n=157)**	**Dominant model**			**Recessive model**		
				**p-value**	**OR**	**RR**	**p-value**	**OR**	**RR**
G43R	127GG	95	144	NC	NC	NC	1.0000		
	127CC	0	0	1.0000			NC	NC	NC
	127GC	9	13						
P141L	422CC	69	116	**0.0177**	**8.524**	**5.399**	0.2115		
	422TT	1	12	0.2115			**0.0177**	**0.117**	**0.1852**
	422CT	34	29						
E162G	485AA	71	111	0.2489			0.6821		
	485GG	1	6	0.6821			0.2489		
	485AG	32	40						
D326Y	976GG	96	66	**<0.0001**	**30.645**	**17.013**	**<0.0001**	**16.545**	**7.333**
	976TT	1	36	**<0.0001**	**0.060**	**0.136**	**<0.0001**	**0.033**	**0.058**
	976GT	7	55						
H451R	1352AA	89	135	NC	NC	NC	1.0000		
	1352GG	0	0	1.0000			NC	NC	NC
	1352AG	15	22						
G484G	1452GG	95	141	NC	NC	NC	0.8306		
	1452AA	0	0	0.8306			NC	NC	NC
	1452GA	9	16						
P574L	1721CC	28	41	0.5039			0.3853		
	1721TT	14	32	0.3853			0.5039		
	1721CT	62	84						
G895G	2685AA	44	76	**0.0399**	**0.336**	**0.589**	0.3752		
	2685CC	11	6	0.3752			**0.0399**	**2.977**	**1.698**
	2685AC	49	75						
***COL4A4* polymorphism**	**Genotype**	**Cases (n=104)**	**Controls (n=157)**	**Dominant model**			**Recessive model**		
				**p-value**	**OR**	**RR**	**p-value**	**OR**	**RR**
P482S	1444CC	31	36	**0.0147**	**0.360**	**0.597**	0.2474		
	1444TT	18	11	0.2474			**0.0147**	**2.788**	**1.674**
	1444CT	55	110						
G545A	1634GG	97	144	NC	NC	NC	0.8130		
	1634CC	0	0	0.8130			NC	NC	NC
	1634GC	7	13						
G789G	2367AA	97	145	0.3985			1.0000		
	2367TT	1	0	1.0000			0.3985		
	2367AT	6	12						
M1327V	3979AA	5	62	0.0897			**<0.0001**	**0.077**	**0.146**
	3979GG	35	37	**<0.0001**	**12.922**	**6.838**	0.0897		
	3979AG	64	58						
V1516V	4548AA	32	53	**0.0362**	**1.993**	**1.561**	0.6861		
	4548GG	17	44	0.6861			**4**	**0.502**	**0.641**
	4548AG	55	60						
F1644F	4932CC	43	46	**0.0038**	**2.890**	**2.053**	**0.0469**	**1.701**	**1.362**
	4932TT	11	40	**0.0469**	**0.588**	**0.734**	**0.0038**	**0.346**	**0.487**
	4932CT	50	71						

Significant differences are shown in bold. Cases: keratoconus patients, Controls: healthy blood donors, n: number of individuals, NC- not calculated, Genotype: Genotypes found representing each polymorphism in cases and controls. Fisher’s exact test was used for statistics. Differences between genotypes are significant when two-sided p-value (p-value) is less than 0.05. OR: Odds ratio, RR: relative risk. OR and RR are shown only for genotypes with significant differences (p-value less than 0.05). Dominant model column shows Fisher’s test results calculated from the sum of the number of individuals with homozygous and heterozygous genotypes compared to the number of individuals with another homozygous genotype for each polymorphism. Recessive model column shows Fisher’s test results obtained by comparing the number of individuals with homozygous genotype against the sum of individuals with another homozygous or heterozygous genotype for each polymorphism.

**Table 6:** Genotype representation and associations under additive model between keratoconus patients and controls.

***COL4A3* polymorphism**	**Genotype**	**Genotype comparison**	**Additive model**		
			**p-value**	**OR**	**RR**
G43R	127GG	GG versus CC	NC	NC	NC
	127CC	GC versus GG	1.0000		
	127GC	GC versus CC	NC	NC	NC
P141L	422CC	**CC versus TT**	**0.0325**	**7.138**	**4.849**
	422TT	**CT versus CC**	**0.0262**	**1.971**	**1.447**
	422CT	**CT versus TT**	**0.0022**	**14.069**	**7.016**
E162G	485AA	AA versus GG	0.2550		
	485GG	AG versus AA	0.4790		
	485AG	AG versus GG	0.2289		
D326Y	976GG	**GG versus TT**	**<0.0001**	**52.364**	**21.926**
	976TT	**GT versus GG**	**<0.0001**	**0.0875**	**0.1905**
	976GT	GT versus TT	0.2520		
H451R	1352AA	AA versus GG	NC	NC	NC
	1352GG	AG versus AA	1.0000		
	1352AG	AG versus GG	NC	NC	NC
G484G	1452GG	GG versus AA	NC	NC	NC
	1452AA	GA versus GG	0.8306		
	1452GA	GA versus AA	NC	NC	NC
P574L	1721CC	CC versus TT	0.3248		
	1721TT	CT versus CC	0.8825		
	1721CT	CT versus TT	0.1686		
G895G	2685AA	**AA versus CC**	**0.0352**	**0.3158**	**0.5667**
	2685CC	AC versus AA	0.6933		
	2685AC	AC versus CC	0.0667		
***COL4A4* polymorphism**	**Genotype**	**Genotype comparison**	**Additive model**		
			**p-value**	**OR**	**RR**
P482S	1444CC	CC versus TT	0.1857		
	1444TT	CT versus CC	0.0726		
	1444CT	**CT versus TT**	**0.0061**	**0.3056**	**0.5370**
G545A	1634GG	GG versus CC	NC	NC	NC
	1634CC	GC versus GG	0.8130		
	1634GC	GC versus CC	NC	NC	NC
G789G	2367AA	AA versus TT	0.4033		
	2367TT	AT versus AA	0.6270		
	2367AT	AT versus TT	0.3684		
M1327V	3979AA	**AA versus GG**	**<0.0001**	**0.0853**	**0.1535**
	3979GG	**AG versus AA**	**<0.0001**	**13.683**	**7.030**
	3979AG	AG versus GG	0.6567		
V1516V	4548AA	AA versus GG	0.2864		
	4548GG	AG versus AA	0.1940		
	4548AG	**AG versus GG**	**0.0153**	**2.373**	**1.716**
F1644F	4932CC	**CC versus TT**	**0.0021**	**3.399**	**2.240**
	4932TT	CT versus CC	0.3283		
	4932CT	**CT versus TT**	**0.0148**	**2.561**	**1.916**

Significant differences are shown in bold. Statistics were based on genotype representation shown in Table 5 (104 keratoconus patients and 157 controls). NC- not calculated, Genotype: Genotypes found representing each polymorphism in cases and controls. Fisher’s exact test was used for statistics. Genotype comparison: genotypes compared against each other. Differences between genotypes are significant when two-sided p-value (p-value) is less than 0.05. OR: Odds ratio, RR: relative risk. OR and RR are shown only for genotypes with significant differences (p-value less than 0.05).

### SIFT and PolyPhen predictions for polymorphisms causing amino acid substitution

The potential impact of polymorphisms causing amino acid substitution was assessed with two analytic tools: SIFT and PolyPhen. SIFT is a sequence homology-based tool that sorts intolerant from tolerant amino acid substitutions and predicts whether an amino acid substitution in a protein will have a phenotypic effect. SIFT is based on the premise that protein evolution is correlated with protein function. Positions important for function should be conserved in the alignment of the protein family, whereas unimportant positions should appear diverse in the alignment. The SIFT tool calculates a score for the amino acid substitution, and a score lower than 0.05 is considered potentially damaging (Table 7). PolyPhen (Brigham and Women's Hospital, Harvard Medical School) is a tool for predicting the possible impact of an amino acid substitution on the structure and function of a human protein. This prediction is based on straightforward empirical rules, which are applied to the sequence, phylogenetic, and structural information characterizing the substitution. The PolyPhen tool uses Position-Specific Independent Counts software to calculate profile scores obtained from the likelihood of a given amino acid occurring at a position of interest compared to background frequencies (the likelihood of this amino acid occurring at any position; Table 7).

**Table 7:** Prediction of effect of substitution polymorphisms found in KC and healthy population.

***COL4A3* polymorphism**	**PolyPhen prediction (score)**	**SIFT prediction (score)**
G43R	possibly damaging (1.800)	not tolerated (0.00)
P141L	probably damaging (2.250)	tolerated (0.40)
E162G	benign(0.024)	tolerated (0.64)
D326Y	probably damaging (2.025)	tolerated (0.08)
H451R	benign (1.426)	tolerated (0.54)
P574L	probably damaging (2.250)	tolerated (0.19)
***COL4A4* polymorphism**	**PolyPhen prediction (score)**	**SIFT prediction (score)**
P482S	benign (1.125)	tolerated (0.71)
G545A	benign (1.350)	not tolerated (0.01)
M1327V	benign(0.017)	tolerated (0.13)

The PolyPhen (Polymorphism Phenotyping) tool predicts the possible impact of an amino acid substitution on the structure and function of a human protein using straightforward physical and comparative considerations. The SIFT (Sorting Intolerant From Tolerant) tool predicts whether an amino acid substitution will affect the protein function; based on sequence homology and the physical properties of amino acids it calculates the potential impact of the amino acid change (score lower than 0.05 is considered potentially damaging).

## Results

### Mutational analysis

Mutational analysis of all exons in *COL4A3* and *COL4A4* genes did not reveal any mutations in KC patients. We detected eight polymorphisms in *COL4A3*, six of them amino substitutions (G43R, P141L, E162G, D326Y, H451R, and P574L), and six polymorphisms in *COL4A4*, three of them amino acid substitutions (P482S, G545A, and M1327V; Table 3, Figure 1 and Figure 2). All of the polymorphisms were also detected in the healthy population and have previously been described as showen in Table 3.

### Hardy-Weinberg equilibrium

When analyzing the H-W equilibrium, we discovered that the frequencies of most of the polymorphisms discovered deviate from expected numbers in both KC patients and controls. In the *COL4A3* gene, only three (P141L, D326Y, and G895G) polymorphisms in the control group and two (D326Y and P574L) polymorphisms in the KC patient group had a p value less than the 5% limit, which was the cut-off value for determining no statistical deviation from the H-W equilibrium. In *COL4A4,* the observed frequencies of three polymorphisms (P428S, M1327V, and V1516V) in the control group and two (G789G and M1327V) in the KC patient group did not deviate from the H-W equilibrium (Table 3).

### Associations between allele and genotype frequencies

The allele frequency in three polymorphisms was significantly associated with KC patients (Table 4). P141L, D326Y, and G895G in *COL4A3* and P482S, M1327V, V1516V, and F1644F in *COL4A4* polymorphisms were associated with KC patients, either as genotypes or alleles, with calculated p values less than 0.05 (Fisher’s exact test; Table 4, Table 5, and Table 6). Significantly represented alleles in KC patients are 976G (D326Y, *COL4A3*) with OR= 15.017, 3979G (M1327V, *COL4A4*) with OR= 2.497, and 4932C (F1644F, *COL4A4*) with OR= 1.750 (Table 4). When analyzing the representation of genotypes for all the polymorphisms found between KC patients and controls, we discovered that some of the genotypes were significantly represented only in the KC patient group (Table 4). The analysis was performed in relation to the representation of mandatory both (recessive) or at least one allele (dominant) being changed.

In terms of the dominant model, genotypes 422CC (OR=8.524) and 976GG (OR=30.645) in *COL4A3* and 3979GG (OR=12.922), 4548AA (OR=1.993), and 4932CC (OR= 2.890) in *COL4A4* are significantly related to KC patients (Table 5). Significantly higher represented genotypes with the recessive model are 976GG (OR=16.545) and 2685CC (OR=2.977) in *COL4A3* and 1444TT (OR=2.788) and 4932CC in *COL4A4* (OR=1.701; Table 5).

We also discovered through analysis of both models that some of the genotypes were significantly less frequent in KC patients: 976TT and 2685AA in *COL4A3* and 1444CC and 4932 TT in *COL4A4* for the dominant model; 422TT and 976TT in *COL4A3* and 3979AA, 4548GG, and 4932TT in *COL4A4* for the recessive model (Table 5). In the additive model, genotypes 422CC, 422TT, 422CT, 976GG, 976TT, and 2685AA in *COL4A3* and 1444CT, 3979AA, 3979GG, 4548AG, 4932CC, and 4932CT in *COL4A4* were significantly different between KC patients and the control group (Table 6).

### SIFT and PolyPhen predictions

PolyPhen analysis predicted that G43R, P141L, D326Y, and P574L polymorphisms in the *COL4A3* gene are potentially damaging. All tested missense polymorphisms in *COL4A4* are predicted to be benign. SIFT tool analysis gave a score less than 0.05 for G43R in *COL4A3* and G545A in *COL4A4*. Those substitutions are predicted to affect the protein function and would not be tolerated. All other substitutions are predicted as tolerated (Table 7).

## Discussion

To our knowledge this is the first report describing the genetic screening of two type IV collagen genes in KC patients. Frequent polymorphisms in affected and healthy populations were found, but no mutations in either of the genes that could be related to KC were discovered. Previous data have revealed that the expression of type IV collagen is deregulated in KC patients and that chromosome locations with genes important in the regulation of collagen synthesis (including type IV collagen) are frequently subjected to aneuploidy and translocation [18,31]. Given the identification of changed amounts of collagen and no affirmative data about relations between mutations in already researched collagen genes and KC, we analyzed the *COL4A3* and *COL4A4* genes, which are deregulated in KC patients, are often subjected to chromosomal aberrations, and could also be responsible for a decrease in collagen types I and III, a feature often detected in the disease [8,9,11,18,19,31].

All of the alterations found in both genes have already been published in other studies. When analyzing whether polymorphisms found were in H-W equilibrium, we discovered that most of them were not. It is difficult to speculate the main reason for this, but some of the probable causes of population differences shown in the study are selection, small population size, population stratification, and genetic drift. It is not rare to find that polymorphisms are in H-W disequilibrium because of the above-mentioned reasons. The control group was selected as described in the Methods section. Considering the small number of some of the alleles found, it is easy to predict that a larger sample size of controls and inclusion of different nationalities and races could help to meet the criteria for H-W equilibrium, but the allele frequencies described in our study are comparable to ones found by Šlajpah et al. [32]. Even though obvious violations to the H-W equilibrium were detected, genotypes and allele representations for some polymorphisms statistically differ between groups and are much more frequent in the KC patients than in the healthy population, which should be taken into consideration when assessing differences between genotypes and phenotypes for a chosen population.

For predicting the effect of substitutions found, we used two different tools, PolyPhen and SIFT, which predict the possible impact on the structure and function of protein substitutions. *COL4A3* G43R, P141L, D326Y, and P574L polymorphisms were predicted to have an effect when analyzed with PolyPhen, but SIFT predicted that only G43R would be damaging. Out of all the substitutions found in *COL4A4*, only G545A was predicted by SIFT to be damaging. Discrepancies between predictions using different tools are expected because the matrices and nature of assessing the damaging effects are based differently. PolyPhen predicts the functional effect of substitutions by determining the level of sequence conservation between homologous genes over evolutionary time, the properties of the exchanged residues, and the proximity of the substitution to predicted putative protein domains and structural features within the protein. SIFT predicts the functional importance of an amino acid substitution based on the alignment of highly similar protein sequences. Predictions rely on whether or not an amino acid at the position of our interest is conserved in the protein family, which can be indicative of its importance to the normal function or structure of the expressed protein. Not all substitutions predicted to affect protein function are involved in disease development and/or progression, especially in the complex diseases, such as KC. Still in the absence of functional data, it is advantageous to use predictive tools to identify substitutions that would more likely affect wild-type protein function; nevertheless differences in results using prediction tools and statistical evaluation of allele/genotype distribution between groups are to be expected.

The allele distributions of three polymorphisms already described in previous studies related to Alport syndrome (D326Y [29] in *COL4A3* and M1327V [22,33] and F1644F [33] in *COL4A4*) were significant for the KC patient cohort. We cannot speculate that these polymorphisms in any way alter the collagen assembly or promote KC disease, although SIFT and PolyPhen predict D326Y to be damaging and the substitution could have an effect on the structure and function of the protein. The other two alleles significant for KC patients were found in the *COL4A4* gene, resulting in one missense and one silent alteration (3979G, M1327V and 4932C, F1644F), although substitution is predicted to be benign and tolerated. When comparing genotypes, we discovered specific genotypes related to KC patients even though the allele distribution was not significantly different. Under different models (dominant, recessive, and additive), we found a significant representation of the following genotypes: 422CC, 422TT, 422CT, and 2685CC in *COL4A3* and 1444TT, 4548AA, and 4548AG in *COL4A4*. The prediction tools used showed the possibility that some of the substitution resulting from these genotypes could be damaging (Table 7). In order to conclude whether genotype representations are specific for our population or are in fact disease specific, different populations should be examined and data compared.

In view of the lack of mutations, we could speculate that mutations in collagen type IV (*COL4A3* and *COL4A4* genes) are not involved in KC disease and that other genes and factors are involved in the pathogenesis of this disorder, but functional assay would be required to clarify this speculation. This study established that significant relationships between KC patients and different genotypes in *COL4A3* and *COL4A4* exist, so the significance of the genotypes should be established by further analysis that would involve different populations. There is a possibility that some of the polymorphisms could be related to KC, a feature that could be used in helping the determination of the molecular genetics of the disease.

## References

[1] Rabinowitz YS Keratoconus. Surv Ophthalmol 1998 42 297 319 9493273 10.1016/s0039-6257(97)00119-7

[2] Héon E Greenberg A Kopp KK Rootman D Vincent AL Billingsley G Priston M Dorval KM Chow RL McInnes RR Heathcote G Westall C Sutphin JE Semina E Bremner R Stone EM VSX1: a gene for posterior polymorphous dystrophy and keratoconus. Hum Mol Genet 2002 11 1029 36 11978762 10.1093/hmg/11.9.1029

[3] McMahon TT Shin JA Newlin A Edrington TB Sugar J Zadnik K Discordance for keratoconus in two pairs of monozygotic twins. Cornea 1999 18 444 51 10422858 10.1097/00003226-199907000-00010

[4] Newsome DA Foidart JM Hassell JR Krachmer JH Rodrigues MM Katz SI Detection of specific collagen types in normal and keratoconus corneas. Invest Ophthalmol Vis Sci 1981 20 738 50 7016805

[5] Wang Y Rabinowitz YS Rotter JI Yang H Genetic epidemiological study of keratoconus: evidence for major gene determination. Am J Med Genet 2000 93 403 9 10951465

[6] Kennedy RH Bourne WM Dyer JA A 48-year clinical and epidemiologic study of keratoconus. Am J Ophthalmol 1986 101 267 73 3513592 10.1016/0002-9394(86)90817-2

[7] Abalain JH Dossou H Colin J Floch HH Levels of collagen degradation products (telopeptides) in the tear film of patients with keratoconus. Cornea 2000 19 474 6 10928761 10.1097/00003226-200007000-00014

[8] Critchfield JW Calandra AJ Nesburn AB Kenney MC Keratoconus: I. Biochemical studies. Exp Eye Res 1988 46 953 63 3197764 10.1016/s0014-4835(88)80047-2

[9] Kenney MC Nesburn AB Burgeson RE Butkowski RJ Ljubimov AV Abnormalities of the extracellular matrix in keratoconus corneas. Cornea 1997 16 345 51 9143810

[10] Maurice DM The structure and transparency of the cornea. J Physiol 1957 136 263 86 13429485 10.1113/jphysiol.1957.sp005758 PMC1358888

[11] Meek KM Tuft SJ Huang Y Gill PS Hayes S Newton RH Bron AJ Changes in collagen orientation and distribution in keratoconus corneas. Invest Ophthalmol Vis Sci 2005 46 1948 56 15914608 10.1167/iovs.04-1253

[12] Hopfer U Fukai N Hopfer H Wolf G Joyce N Li E Olsen BR Targeted disruption of Col8a1 and Col8a2 genes in mice leads to anterior segment abnormalities in the eye. FASEB J 2005 19 1232 44 16051690 10.1096/fj.04-3019com

[13] Aldave AJ Bourla N Yellore VS Rayner SA Khan MA Salem AK Sonmez B Keratoconus is not associated with mutations in COL8A1 and COL8A2. Cornea 2007 26 963 5 17721297 10.1097/ICO.0b013e31811dfaf7

[14] Biswas S Munier FL Yardley J Hart-Holden N Perveen R Cousin P Sutphin JE Noble B Batterbury M Kielty C Hackett A Bonshek R Ridgway A McLeod D Sheffield VC Stone EM Schorderet DF Black GC Missense mutations in COL8A2, the gene encoding the alpha2 chain of type VIII collagen, cause two forms of corneal endothelial dystrophy. Hum Mol Genet 2001 10 2415 23 11689488 10.1093/hmg/10.21.2415

[15] Gottsch JD Sundin OH Liu SH Jun AS Broman KW Stark WJ Vito EC Narang AK Thompson JM Magovern M Inheritance of a novel COL8A2 mutation defines a distinct early-onset subtype of fuchs corneal dystrophy. Invest Ophthalmol Vis Sci 2005 46 1934 9 15914606 10.1167/iovs.04-0937

[16] Määttä M Heljasvaara R Sormunen R Pihlajaniemi T Autio-Harmainen H Tervo T Differential expression of collagen types XVIII/endostatin and XV in normal, keratoconus, and scarred human corneas. Cornea 2006 25 341 9 16633037 10.1097/01.ico.0000178729.57435.96

[17] Määttä M Väisänen T Väisänen MR Pihlajaniemi T Tervo T Altered expression of type XIII collagen in keratoconus and scarred human cornea: Increased expression in scarred cornea is associated with myofibroblast transformation. Cornea 2006 25 448 53 16670484 10.1097/01.ico.0000183537.45393.1f

[18] Bochert A Berlau J Koczan D Seitz B Thiessen HJ Guthoff RF Gene expression in keratoconus. Initial results using DNA microarrays. Ophthalmologe 2003 100 545 9 12920555 10.1007/s00347-003-0808-0

[19] Stachs O Bochert A Gerber T Koczan D Thiessen HJ Guthoff RF The extracellular matrix structure in keratoconus. Ophthalmologe 2004 101 384 9 15067420 10.1007/s00347-003-0902-3

[20] Mariyama M Zheng K Yang-Feng TL Reeders ST Colocalization of the genes for the alpha-3(IV) and alpha-4(IV) chains of type IV collagen to chromosome 2 bands q35-q37. Genomics 1992 13 809 13 1639407 10.1016/0888-7543(92)90157-n

[21] Momota R Sugimoto M Oohashi T Kigasawa K Yoshioka H Ninomiya Y Two genes, COL4A3 and COL4A4 coding for the human alpha-3(IV) and alpha-4(IV) collagen chains are arranged head-to-head on chromosome 2q36. FEBS Lett 1998 424 11 6 9537506 10.1016/s0014-5793(98)00128-8

[22] Boye E Mollet G Forestier L Cohen-Solal L Heidet L Cochat P Grünfeld JP Palcoux JB Gubler MC Antignac C Determination of the genomic structure of the COL4A4 gene and of novel mutations causing autosomal recessive Alport syndrome. Am J Hum Genet 1998 63 1329 21 9792860 10.1086/302106 PMC1377543

[23] Turner N Mason PJ Brown R Fox M Povey S Rees A Pusey CD Molecular cloning of the human Goodpasture antigen demonstrates it to be the alpha-3 chain of type IV collagen. J Clin Invest 1992 89 592 601 1737849 10.1172/JCI115625 PMC442892

[24] Hudson BG Reeders ST Tryggvason K Type IV collagen: structure, gene organization, and role in human diseases. Molecular basis of Goodpasture and Alport syndromes and diffuse leiomyomatosis. J Biol Chem 1993 268 26033 6 8253711

[25] Mochizuki T Lemmink HH Mariyama M Antignac C Gubler MC Pirson Y Verellen-Dumoulin C Chan B Schroder CH Smeets HJ Reeders ST Identification of mutations in the alpha-3(IV) and alpha-4(IV) collagen genes in autosomal recessive Alport syndrome. Nat Genet 1994 8 77 81 7987396 10.1038/ng0994-77

[26] Lemmink HH Schroder CH Monners LAH Smeets HJM The clinical spectrum of type IV collagen mutations. Hum Mutat 1997 9 477 99 9195222 10.1002/(SICI)1098-1004(1997)9:6<477::AID-HUMU1>3.0.CO;2-#

[27] Jun AS Liu SH Koo EH Do DV Stark WJ Gottsch JD Microarray analysis of gene expression in human donor corneas. Arch Ophthal 2001 119 1629 34 11709013 10.1001/archopht.119.11.1629

[28] Krafchak CM Pawar H Moroi SE Sugar A Lichter PR Mackey DA Mian S Nairus T Elner V Schteingart MT Downs CA Kijek TG Mutations in TCF8 cause posterior polymorphous corneal dystrophy and ectopic expression of COL4A3 by corneal endothelial cells. Am J Hum Genet 2005 77 694 708 16252232 10.1086/497348 PMC1271382

[29] Heidet L Arrondel C Forestier L Cohen-Solal L Mollet G Gutierrez B Stavrou C Gubler MC Antignac C Structure of the human type IV collagen gene COL4A3 and mutations in autosomal Alport syndrome. J Am Soc Nephrol 2001 12 97 106 11134255 10.1681/ASN.V12197

[30] Heukeshoven J Dernick R Native horizontal ultrathin polyacrylamide gel electrophoresis of proteins under basic and acidic conditions. Electrophoresis 1992 13 654 9 1281089 10.1002/elps.11501301137

[31] Pettenati MJ Sweatt AJ Lantz P Stanton CA Reynolds J Rao PN Davis RM The human cornea has a high incidence of acquired chromosome abnormalities. Hum Genet 1997 101 26 9 9385364 10.1007/s004390050580

[32] Šlajpah M Gorinšek B Berginc G Vizjak A Ferluga D Hvala A Meglič A Jakša I Furlan P Gregorič A Kaplan-Pavlovčič Š Ravnik-Glavač M Glavač D Sixteen novel mutations indentified in COL4A3, COL4A4 and COL4A5 genes in Slovenian families with Alport syndrome and benign familial hematuria. Kidney Int 2007 71 1287 95 17396119 10.1038/sj.ki.5002221

[33] Longo I Porcedda P Mari F Giachino D Meloni I Deplano C Brusco A Bosio M Massella L Lavoratti G Roccatello D Frascá G Mazzucco G Muda AO Conti M Fasciolo F Arrondel C Heidet L Renieri A De Marchi M COL4A3/COL4A4 mutations: from familial hematuria to autosomal-dominant or recessive Alport syndrome. Kidney Int 2002 61 1947 56 12028435 10.1046/j.1523-1755.2002.00379.x

[34] Wang YY Rana K Tonna S Lin T Sin L Savige J COL4A3 mutations and their clinical consequences in thin basement membrane nephropathy (TBMN) Kidney Int 2004 65 786 90 14871398 10.1111/j.1523-1755.2004.00453.x

